# Further dissection of QTLs for salt-induced stroke and identification of candidate genes in the stroke-prone spontaneously hypertensive rat

**DOI:** 10.1038/s41598-018-27539-2

**Published:** 2018-06-20

**Authors:** Kaoru Niiya, Hiroki Ohara, Masato Isono, Abdullah Md. Sheikh, Hiroyuki Matsuo, Koichi Fujikawa, Minoru Isomura, Norihiro Kato, Toru Nabika

**Affiliations:** 1 Department of Functional Pathology, Shimane University Faculty of Medicine, Izumo, Japan; 2 Department of Laboratory Medicine, Shimane University Faculty of Medicine, Izumo, Japan; 3 Department of Gene Diagnostics and Therapeutics, Research Institute, National Center for Global Health and Medicine, Tokyo, Japan; 4 Shimane University Faculty of Human Sciences, Matsue, Japan

## Abstract

We previously revealed that two major quantitative trait loci (QTLs) for stroke latency of the stroke-prone spontaneously hypertensive rat (SHRSP) under salt-loading were located on chromosome (Chr) 1 and 18. Here, we attempted further dissection of the stroke-QTLs using multiple congenic strains between SHRSP and a stroke-resistant hypertensive rat (SHR). Cox hazard model among subcongenic strains harboring a chromosomal fragment of Chr-1 QTL region showed that the most promising region was a 2.1 Mbp fragment between D1Rat177 and D1Rat97. The QTL region on Chr 18 could not be narrowed down by the analysis, which may be due to multiple QTLs in this region. Nonsynonymous sequence variations were found in four genes (*Cblc*, *Cxcl17*, *Cic*, and *Ceacam 19*) on the 2.1 Mbp fragment of Chr-1 QTL by whole-genome sequence analysis of SHRSP/Izm and SHR/Izm. Significant changes in protein structure were predicted in CBL-C and CXCL17 using I-TASSER. Comprehensive gene expression analysis in the kidney with a cDNA microarray identified three candidate genes (*LOC102548695* (Zinc finger protein 45-like, *Zfp45L*), *Ethe1*, and *Cxcl17*). In conclusion, we successfully narrowed down the QTL region on Chr 1, and identified six candidate genes in this region.

## Introduction

Cerebral stroke is a major health problem in Japan as well as in the world not only because it is a major cause of death but also because it is a major cause of severe disability in the elderly^1^. Because the stroke-prone spontaneously hypertensive rat (SHRSP) is thought to be a good model for cerebral hemorrhage and lacunar infarction, genetic analysis of this model may provide new insights about the genetic risks of these disorders, which may be useful for the prevention of the disease in humans^2–5^.

We performed a quantitative trait locus (QTL) analysis on stroke susceptibility in F2 progenies between SHR and SHRSP and identified two major QTLs for stroke latency under salt-loading on chromosome (Chr) 1 and 18^6^. Analysis of the double congenic strains for these two QTLs showed that they had an additive effect on stroke latency, which explained a major part of the stroke susceptibility in SHRSP^6^.

Because the QTL regions identified on Chr 1 and 18 were too large for further analysis (about 36 and 37 Mbp, respectively), we attempted to reduce the QTL regions using multiple subcongenic strains in this study. Furthermore, we identified six candidate genes in the Chr-1 QTL region through comprehensive analysis of whole-genome sequence and gene expression.

## Results

### The double subcongenic strain SHRSPrch1.1_18.0

**Figure 1:**
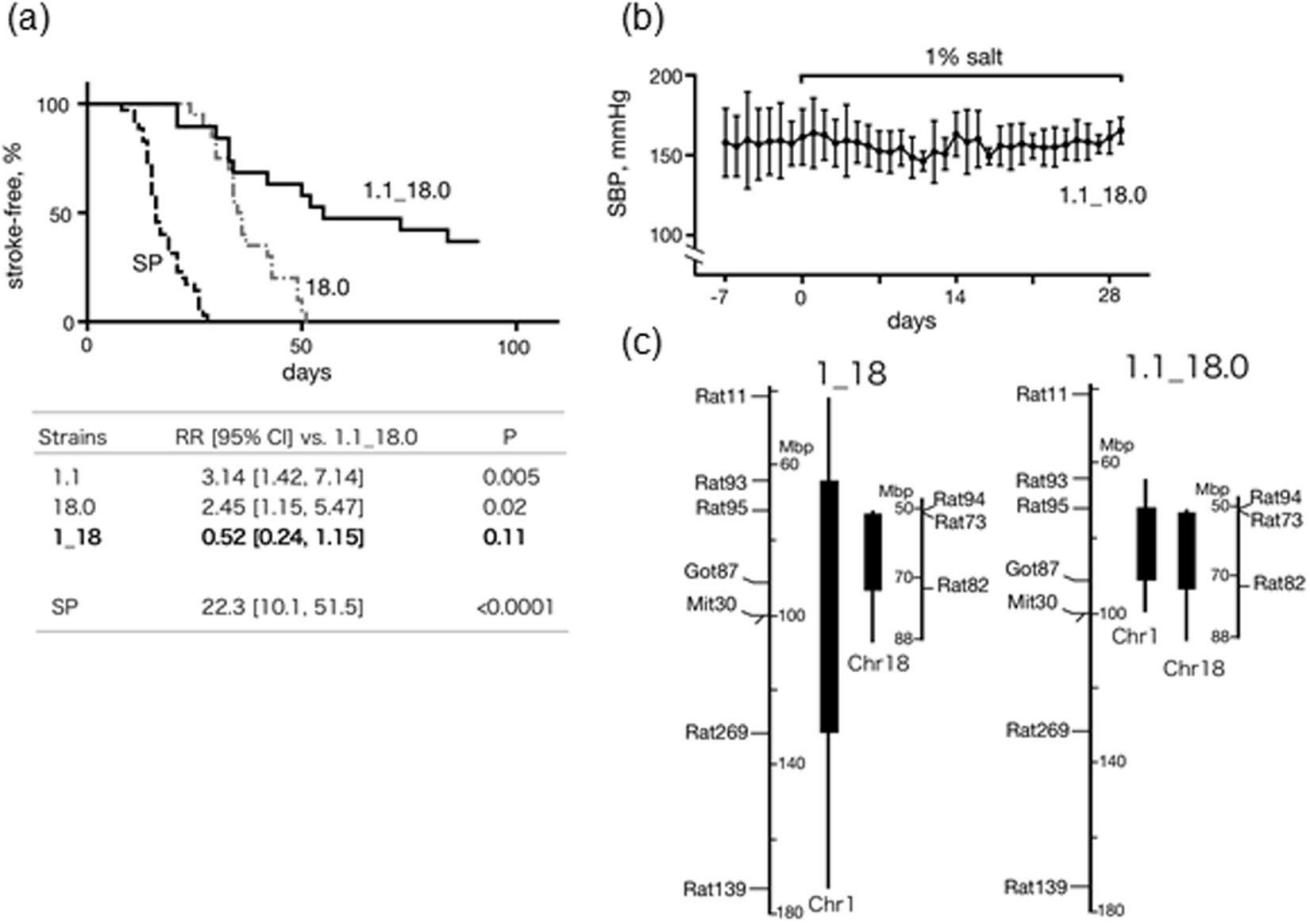
The double subcongenic strain SHRSPrch1.1_18.0. (**a**) Stroke latency under salt-loading. The Cox hazard model was used to calculate relative risks (RR) in the table below. To compare with the latency of SHRSPrch1.1 and 1_18, the data obtained in the previous study were used for these two strains (see ref.^6^). Note that the data newly obtained in this study is shown for SHRSPrch18.0 (see also ref.^6^). 1.1_18.0: SHRSPrch1.1_18.0. (**b**) SBP measured by telemetry during salt-loading. No significant increase was observed (n = 3). (**c**) Genomic construction of SHRSPrch1.1_18.0 in comparison with the original double congenic strain SHRSPrch1_18. Note that the congenic region on Chr 1 was much shorter in SHRSPrch1.1_18.0.

**Figure 2:**
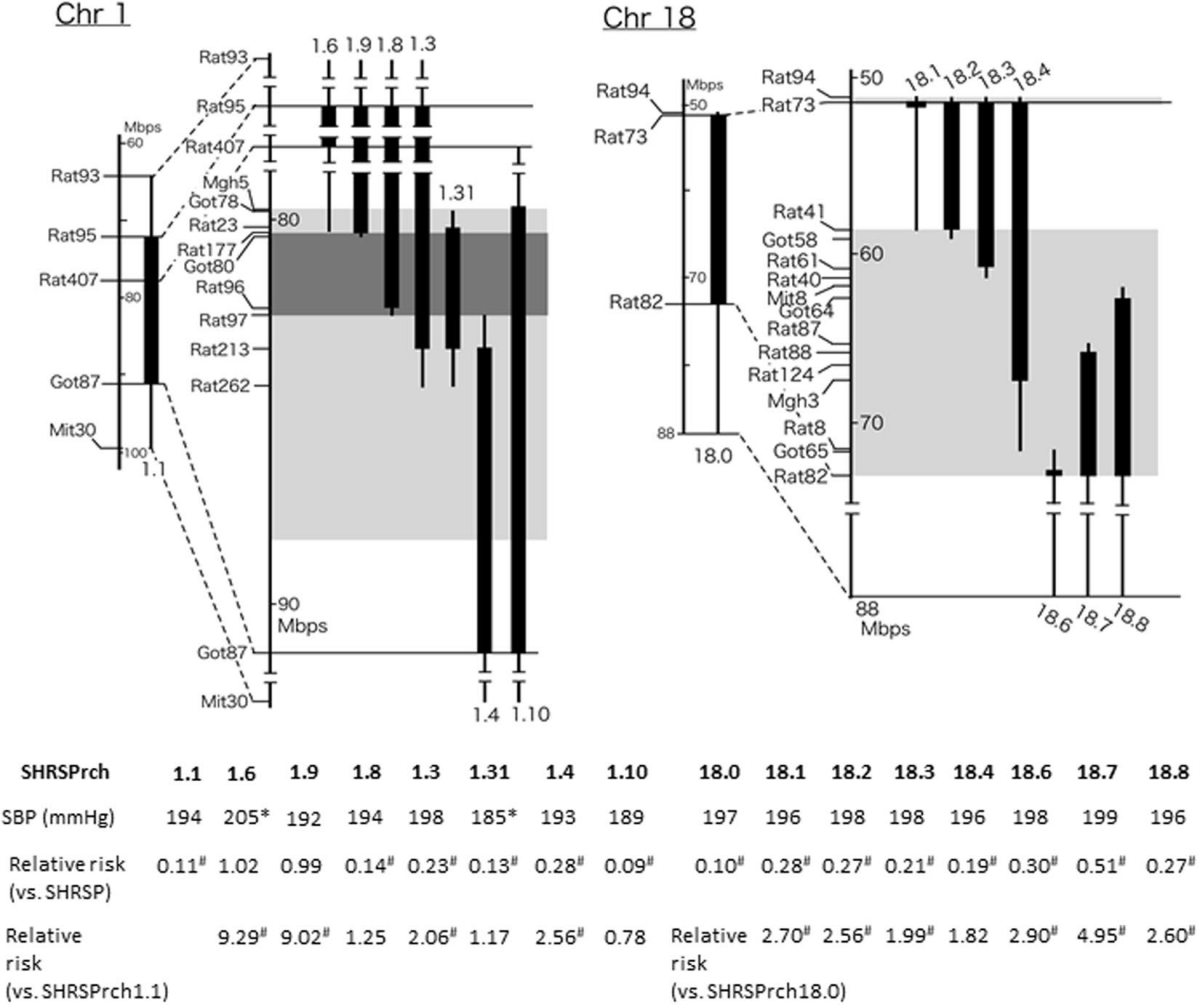
Genomic composition of the subcongenic strains. Congenic regions (homozygous for SHR alleles) of subcongenic strains are shown as closed columns. Vertical bars indicate regions including a recombination. Light grey boxes indicate the haplotype blocks that differed between SHRSP and SHR (see ref.^6^). A dark grey box indicates the region narrowed down through the current analysis of subcongenic strains (see Results). Note that ‘SHRSPrch’ was omitted from the names of the subcongenic lines. ‘SHRSPrch1.’ and ‘SHRSPrch18.’ mean Chr-1 and −18 subcongenic strains, respectively. The numbers following period, e.g., 31 in the SHRSPrch1.31, are the identification number for each subconogenic strain. Averages of systolic blood pressure (SBP) and relative risk of stroke are shown under the columns representing the subcongenic strains (see Table S1 and Fig. 3 for the details). *P < 0.05 vs. SHRSP by Dunnet’s post-hoc test. ^#^*P* < 0.05 by the Cox hazard model.

In the previous study^6^, we showed that a 18 Mbp fragment on Chr1 and a 29 Mbp fragment on Chr18, covered by SHRSP-based congenic strain SHRSPrch1.1 and SHRSPrch18.0 respectively, harbored major genes responsible for the stroke susceptibility under salt-loading in SHRSP. In the present study, we first constructed a new double subcongenic strain, SHRSPrch1.1_18.0, by a cross between SHRSPrch1.1 and SHRSPrch18.0 to confirm whether stroke susceptibility of this strain is comparable to that of the original double congenic strain, SHRSPrch1_18 (Fig. 1c, see also ref.^6^). SHRSPrch1.1_18.0 has the largest chromosomal fragments from the QTL region among the series of subcongenic strains evaluated in this study (see Fig. 1c and Fig. 2). Note that the telomeric side marker on Chr18, D18Rat11 (see ref.^6^), was replaced with D18Rat82 in the present study because of an additional genotyping for this simple sequence repeat marker (Fig. 1c).

As shown in Fig. 1a, the stroke latency in SHRSPrch1.1_18.0 did not significantly differ from that in the original double congenic strain SHRSPrch1_18, but did significantly differ from that in the single congenic strains, SHRSPrch1.1 and SHRSPrch18.0, as well as that in SHRSP. Furthermore, telemetry analysis indicated that no significant systolic blood pressure (SBP) increase was observed under salt-loading in this strain (Fig. 1b), which was similar to that of SHRSPrch1_18^6^. These observations strongly suggested that the regions covered by SHRSPrch1.1_18.0 included the gene(s) for stroke latency as well as salt-induced BP increase that the original double congenic strains harbored. Accordingly, we focused on these regions for further dissection using subcongenic strains.

### Blood pressure and body weight of subcongenic strains

**Figure 3:**
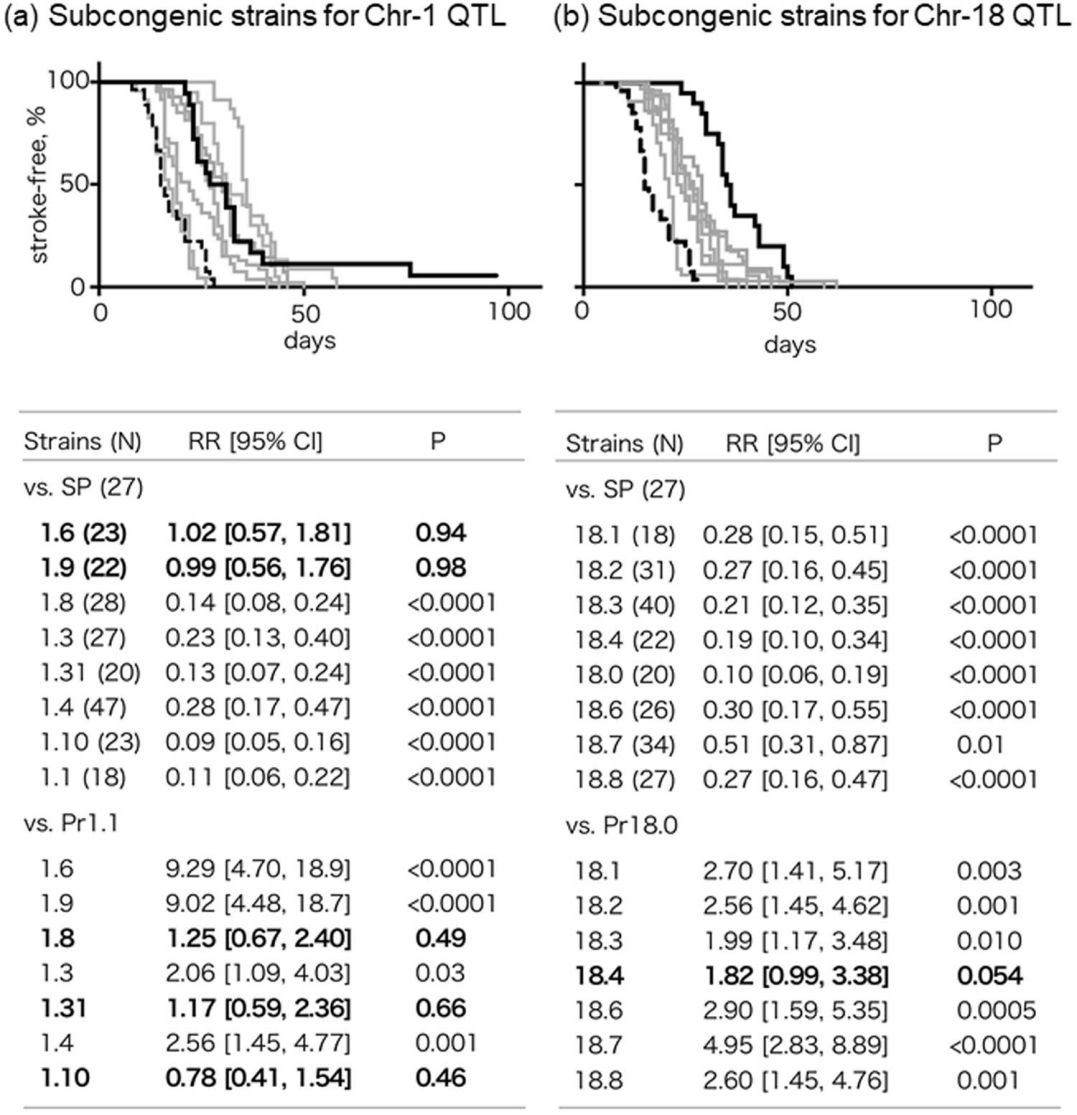
Stroke latency under salt-loading in the subcongenic strains. Stroke latencies of subcongenic strains for the Chr-1 and Chr 18-QTLs are shown on the left (a) and the right (b) panel, respectively. Black lines indicate event-free survival curves for SHRSPrch1.1 (**a**) and 18.0 (**b**). Black broken lines indicate that of SHRSP in both panels. In the lower tables, the strains with bold characters did not show a significant difference in stroke latency from the reference strains. The Cox hazard model was used in the analysis. SP: SHRSP, Pr1.1: SHRSPrch1.1, Pr18.0: SHRSPrch18.0.

SBP and body weight (BW) for the subcongenic strains are shown in Table S1. No significant differences were observed in SBP in Chr-18 subcongenic strains. In contrast, among Chr-1 subcongenic strains, SHRSPrch1.6 and 1.31 had significantly higher and lower SBP than that of SHRSP, respectively. Figure 2 shows genomic composition of the subcongenic strains with their SBP (see also Table S1) and relative risk of stroke (see also Fig. 3). All of them were established by a backcross of SHRSPrch1.1 or 18.0 to SHRSP, i.e., each subcongenic strain has a dissected Chr-1 or −18 QTL fragment from SHRSPrch1.1 or 18.0 (see also Methods).

### Stroke latency of subcongenic strains

Figure 3 summarizes the results of the evaluation of stroke latency in the subcongenic strains. Using the Cox hazard model, we compared stroke latency of the subcongenic strains with that of SHRSP and of SHRSPrch1.1 or 18.0, which had the largest fragment from the QTL region and showed the longest stroke latency among the subcongenic strains examined. As for the QTL on Chr 1, the analysis on the subcongenic strains indicated that the latency in SHRSPrch1.8, 1.31, and 1.10 did not significantly differ from that in SHRSPrch1.1. When compared with SHRSP, the two subcongenic strains, SHRSPrch1.6 and 1.9, showed stroke latency that was not different from that in SHRSP, indicating that the region harbored by these two subcongenic strains could be excluded from the target region. SBP had a marginally significant effect on stroke latency (*P* = 0.03). These observations implied that the most promising region was the 2.1 Mbp fragment between D1Rat177 and D1Rat97 (see Fig. 2). The data from SHRSPrch1.3 may need more careful interpretation. The relative risk to SHRSPrch1.1 was marginally significant in this congenic strain even though it harbored the target region above. This suggests that SHRSPrch1.3 has an additional genetic locus (or loci) with an adverse impact on the stroke latency.

In contrast to the Chr-1 QTL, it was difficult to narrow down the QTL region on Chr 18 by analysis of the subcongenic strains – stroke latency in most of the subcongenic strains differed significantly from both SHRSP and SHRSPrch18.0, which may be a result of multiple QTLs in this region (see Discussion). SBP did not show a significant effect on the stroke latency among the subcongenic strains for the Chr-18 QTL (*P* = 0.10). Similar reasons to SHRSPrch1.3 may apply to the data for SHRSPrch18.3, 18.4, and 18.7. These strains had marginal levels of significance in risk relative to the reference strains. The genomic fragments harbored by these congenic strains may need careful evaluation.

### Identification of candidate genes by gene expression and sequencing analysis

**Figure 4:**
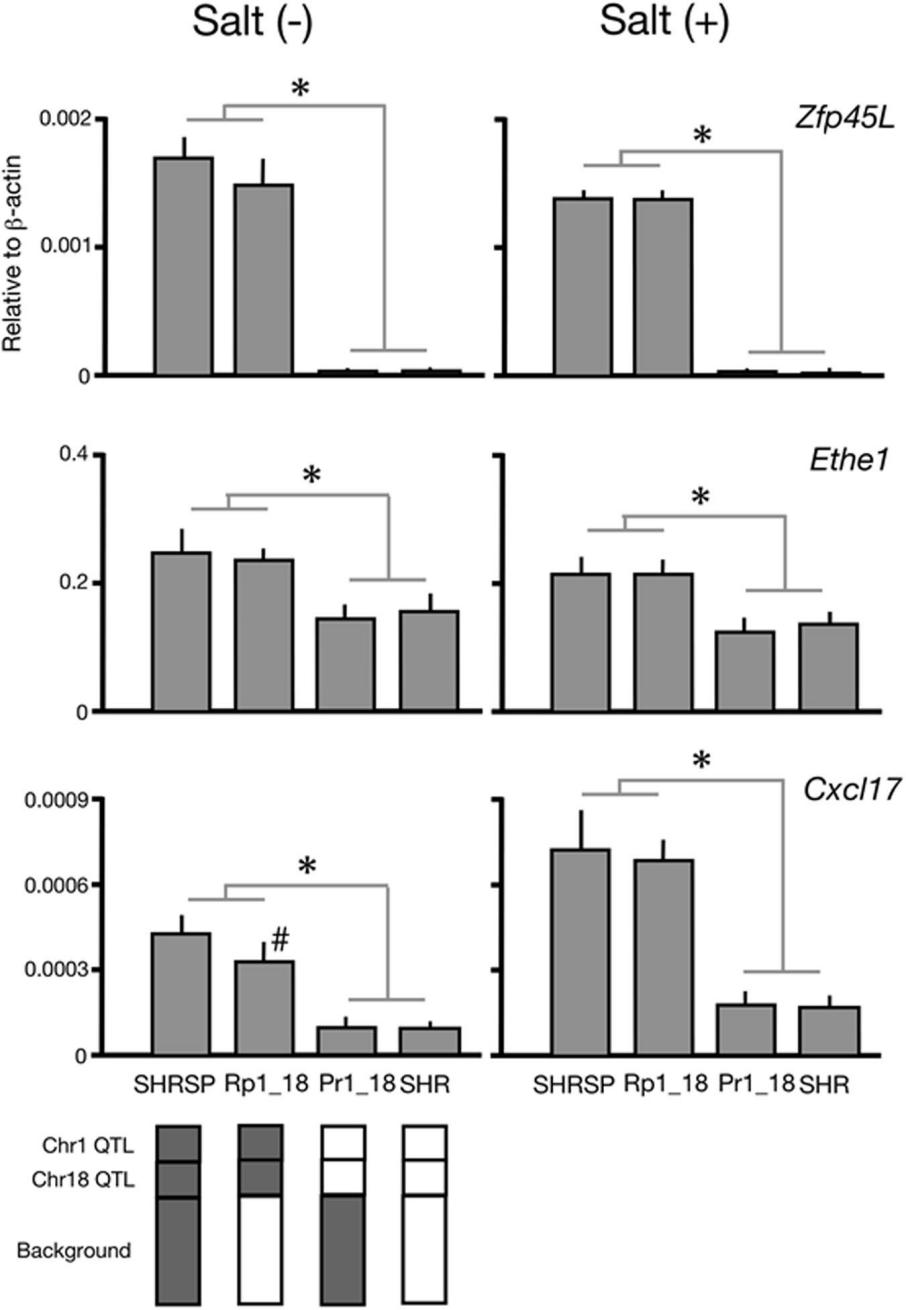
Expression of three candidate genes in the kidney. Kidneys were collected from rats with [Salt (+)] and without [Salt (−)] salt-loading (1% salt water for 1 week). Quantitative RT-PCR was performed as described previously (ref.^28^, see also Methods). SHRSPrch1_18 and SHRpch1_18 were abbreviated as Pr1_18 and Rp1_18, respectively. All three genes showed a significantly lower expression in both Pr1_18 and SHR than in Rp1_18 and SHRSP (*), which was consistent with the hypothetical expression pattern (see Results). In addition, a significant difference was observed between SHRSP and Rp1_18 (#) in *Cxcl17* without salt-loading. Tukey’s multiple comparison test was employed in the analysis. Samples from five rats of each strain were used. Schematic patterns of genomic composition of the four strains are shown in the bottom of the figure; grey and open boxes indicate the genome from SHRSP and SHR, respectively.

Because it was difficult to deduce the small target region on Chr 18, we focused on the 2.1 Mbp region on Chr 1. To explore candidate genes in this region, we performed comprehensive gene expression analysis using two parental strains, SHR and SHRSP, and two ‘reciprocal’ double congenic strains, SHRSPrch1_18 and SHRpch1_18. Because telemetry data implies that a salt-induced BP increase has a key role in the stroke susceptibility in congenic rats (SHRpch1_18 showed a clear salt-sensitive BP increase but SHRSPrch1_18 did not, see ref.^6^), we focused on gene expression in the kidney first. To identify genes whose expression is regulated by Chr-1 and Chr-18 QTLs, we extracted genes whose expression level depended on the genotype of the QTL regions (i.e., the expression level differed between SHRSP/SHRpch1_18 and SHR/SHRSPrch1_18, see the bottom of Fig. 4).

**Figure 5:**
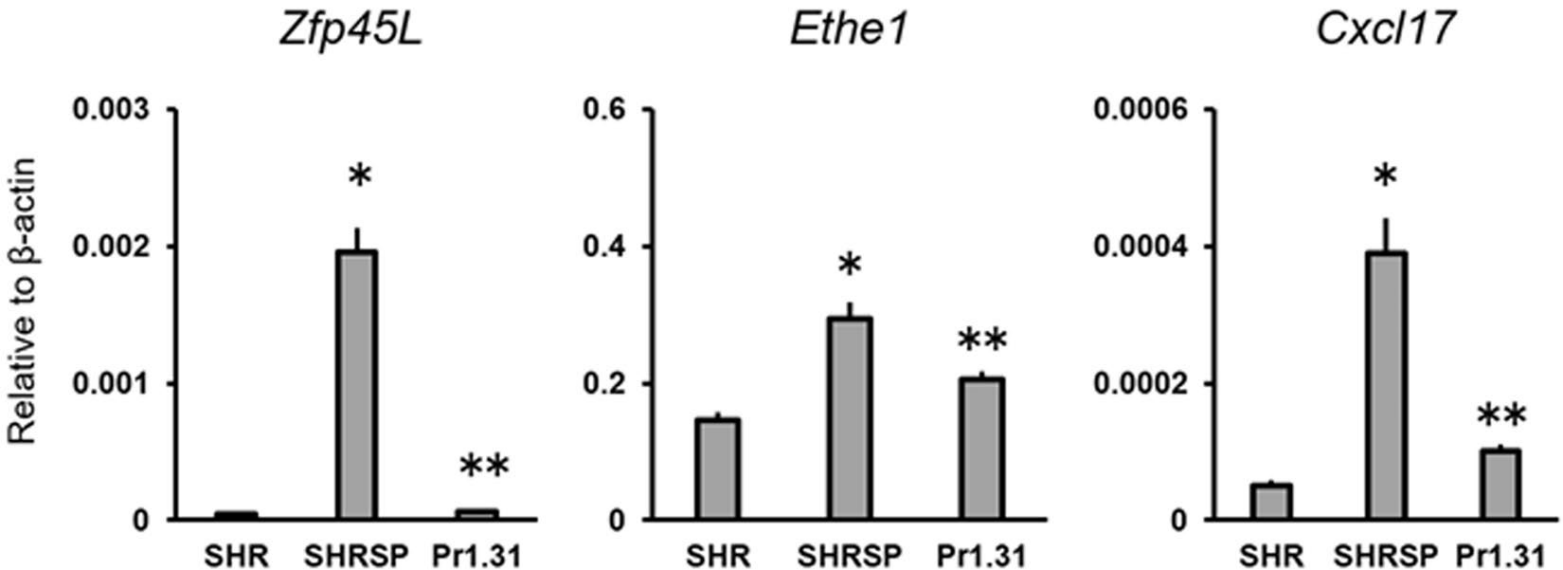
Gene expression of the three candidate genes in SHRSPrch1.31. Kidneys were collected from the three rat strains at 12–14 weeks of age without salt-loading. Total RNA was extracted and quantitative RT-PCR was performed as previously described (ref.^28^, see also Methods in the main text). Pr1.31; SHRSPrch1.31, *P < 0.05 vs. SHR, **P < 0.05 vs. SHRSP by Student’s t-test. Five rats were used for each strain.

In the target region on Chr 1, 114 genes were located. Among them, we identified three genes, *LOC102548695* (Zinc finger protein 45-like, hereinafter referred to as *Zfp45L*)*, Ethe1*, and *Cxcl17*, that fulfilled the hypothetical expression pattern from the four strains under baseline conditions (see Fig. S1). These genes all had a similar pattern of expression, even under salt-loading (Fig. S1). We confirmed this expression pattern by quantitative RT-PCR under the baseline as well as under the salt-loaded condition (Fig. 4). Expression of the three genes was significantly greater in the strains with the SHRSP allele of the QTL regions. We further confirmed that the expression of these three genes was regulated by the allele of the Chr-1 QTL using SHRSPrch1.31 (Fig. 5).

Additional screening was performed on the whole-genome sequence data from SHRSP/Izm and SHR/Izm. We identified five missense variations in the coding sequence of four genes, which were confirmed by direct sequencing (see Table 1). No nonsense variations nor frame-shift variations were identified in the genes located in this region. Conformation analysis of the protein products of these genes using I-TASSER implied that the variations in CBL-C and CXCL17 might cause significant changes in the protein structure (see Fig. S2). Furthermore, we performed direct sequencing in nine popular strains of laboratory rats to confirm whether the variations in *Cblc* and *Cxcl17* were specific for SHRSP. As shown in Table S2, the variations seemed specific for the SHRSP strain, but DON/Kyo (NBRP Rat No.0004, a standard albino strain with Japan origin) also had the same variations as SHRSP. The variation in *Ceacam 19* is located in the transmembrane portion, which seems to cause no major changes in the conformation (data not shown). *Cic* could not be analyzed because there was no information in the PDB.

**Table 1:** Missense variations in coding sequence between SHR and SHRSP in the target region.

Gene	ID	position in CDS	SHR	SHRSP
Cbl proto-oncogene C (*Cblc*)	NM_001034920	1357 (exon 9)	A (Asn)	G (Asp)
	NM_001034920	977 (exon 6)	T (Val)	A (Glu)
CEA-related cell adhesion molecule 19 (*Ceacam 19*)	NM_001198970	472 (exon 3)	A (Thr)	G (Ala)
Capicua transcriptional repressor (*Cic*)	NM_001107490	2496 (exon 10)	G (Gln)	T (His)
CXC motif chemokine ligand 17 (*Cxcl17*)	NM_001107491	346 (exon 4)	A (Thr)	G (Ala)

## Discussion

In this study, we attempted to narrow down the QTL regions for stroke latency in SHRSP both on Chr 1 and 18, and identified a target region on Chr 1, which was the 2.1 Mbp fragment between D1Rat177 and D1Rat 97. Through comprehensive gene expression analysis and whole-genome sequence analysis, we identified six candidate genes in this region.

In the evaluation process of subcongenic strains for the Chr-18 QTL, the stroke latency in most of the strains was between the shortest (SHRSP) and the longest latency (SHRSPrch18.0). This might be due to multiple QTLs affecting the stroke latency in this region as suggested by the broad peak observed in this QTL^6^. Similar observations were commonly observed in attempts to narrow down QTL regions using subcongenic rats^7–9^.

In contrast, analysis on subcongenic strains for the Chr-1 QTL strongly suggested that the fragment between D1Rat177 and D1Rat97, shared not by SHRSPrch1.9 but by SHRSPrch1.10, 1.8 and 1.31, was the most likely to harbor the gene(s) affecting stroke latency. However, careful consideration may be necessary for the region covered by SHRSPrch1.4 because this congenic strain showed a significantly lower risk for stroke when compared with that of SHRSP. This region might harbor additional genes influencing stroke latency.

In the new target region, we still have 114 genes (90 protein-coding genes, 15 non-coding RNA, and 9 pseudogenes). To identify candidate genes in this region, we employed comprehensive gene expression analysis as well as comparison of coding sequences using whole-genome sequence data. Through comprehensive gene expression analysis, we expected that many genes with false positive differences might disrupt further analysis. To avoid this problem, we took a unique strategy to use two ‘reciprocal’ double congenic strains, SHRSPrch1_18 and SHRpch1_18, in addition to the parental strains, SHR and SHRSP. We hypothesized that genes whose expression differed significantly between SHR/SHRSPrch1_18 (the QTL regions were from SHR) and SHRSP/SHRpch1_18 (the QTL regions were from SHRSP) were good candidates. Following this criterion, we identified three candidate genes, *Zfp45L*, *Ethe1*, and *Cxcl17*, in this region. Because similar differences were observed even under salt-loading (see Fig. 4), these inter-strain gene expression differences may be constitutively expressed. Interestingly, expression of *Cxcl17* was potentiated under salt-loading only in the strains with SHRSP-derived QTL regions (i.e., SHRSP and SHRpch1_18, see Fig. 4).

It is likely that the Chr1 QTL directly regulates the expression of the three candidate genes (Fig. 5), however, the molecular mechanisms remain unclear. ZFP45L, a putative transcriptional regulator, might control the expression of *Ethe1* and/or *Cxcl17* (see below). Genetic (e.g. nucleotide substitution/deletion/insertion) and/or epigenetic (e.g. CpG island hypermethylation) variations in the promoter region of the genes are also thought to be possible mechanisms.

CXCL17 is a newly identified chemokine expressed mainly in the mucosa of the gut and lung^10,11^. It was reported to induce VEGF expression and angiogenesis as well as macrophage chemotaxis^10,11^. Furthermore, the receptor for CXCL17, GPR35, has been implicated in pathogenesis of hypertension, coronary artery diseases, and cardiac hypertrophy^12–14^. In addition to differences in gene expression, *Cxcl17* in SHRSP had a sequence variation in the coding region and was therefore expected to have a shorter α-helix loop at the C-terminal end (see Results and Fig. S2). Nonetheless, no reports were available on its physiological roles in the kidney and further analysis is essential to elucidate the functional importance of this interesting candidate gene.

ETHE1 is a metalloprotein from the β-lactamase family that is involved in sulfide oxidation in mitochondria^15^. Null mutations in this gene cause the autosomal recessive disorder ethylmalonic encephalopathy (EE) through intracellular accumulation of sulfide^15^. Because microangiopathy was found in patients with EE, a decrease in ETHE1 activity might be harmful for arterial integrity^15^. However, the expression pattern of ETHE1 did not support a role in stroke susceptibility because the expression of *Ethe1* was significantly greater (not lower) in strains with a higher risk for stroke (i.e., SHRSP and SHRpch1_18) (Fig. 4).

Expression of *Zfp45L* was almost null in strains with a lower stroke risk (i.e., SHR and SHRSPrch1_18). Because the zinc-finger family proteins are thought to function as a transcriptional regulator, genes regulated by *Zfp45L* might be causally related to stroke. Although the human ortholog *ZNF45* was reported to have an interaction with C/EBP^16^, little information is available about which genes are regulated by *ZNF45* and the physiological roles of this putative transcription regulator. Through comparison of coding sequences, we identified five missense variations between SHR and SHRSP. A web-based analysis implied that two variations in *Cblc* might result in a substantial conformational change in the protein (Fig. S2). CBLC is a member of the CBL family of proteins, which are ubiquitin ligases that regulate signaling through tyrosine kinase-coupled receptors^17^. The functional roles of CBLC in stroke-susceptibility in SHRSP and the functional importance of the variations inside this gene should be evaluated in future studies.

In addition to established genes in the target region, recent advances in the study of disease mechanisms revealed new regulatory systems of gene expression by non-coding RNAs situated far away from the gene^18,19^. Two microRNAs, miR-330 and miR-343, were found in the candidate region by screening miRNA databases (miRBase and Rat Genome Database), but the expression of these microRNAs was not detected in the kidneys of SHRSP and SHR (data not shown, see Methods). Careful investigation of the possible roles of non-coding RNAs or ‘intergenic’ sequences is essential in future studies, but it is more pragmatic to focus on the six genes listed in this study first because genome editing technologies can be used to elucidate the physiological roles of these genes^20^.

As a limitation in this study, we performed gene expression analysis only in the kidney. Because it has been expected that Chr-1 and −18 QTL affected to salt-induced BP changes in congenic strains (6, see also Fig. 1b), we focused on the kidney whose dysfunction plays a pathologically pivotal role in salt-sensitive hypertension. However, it is of note that Stanzione *et al*. recently reported that decrease in expression of brain miR-122, which exists on the Chr-18 QTL region, is an early marker of cerebral stroke in SHRSP under salt-loading^21^. Given the complexity of pathological mechanisms of cardiovascular diseases, we may need to perform comprehensive gene expression analysis in other organs including the brain.

In summary, we successfully narrowed down the QTL region on Chr 1, and identified six candidate genes in this region through a combined approach using subcongenic analysis, gene expression analysis in the kidney and whole-genome sequencing. Recent advances in Genome Wide Association Studies (GWAS) have identified genetic variations and possible genetic mechanisms in the onset of hypertension and cerebral stroke in human^22–25^. As far as we know, in both hypertension and cerebral stroke, there is no overlap between our candidate genes in rats and GWAS hits in human. However, it seems that common pathological mechanisms, such as imbalanced inflammatory responses^26^, play a role in the onset of those diseases in both human and rodents. Regarding this point, CXCL17 may be a possible candidate in the present study, because this novel cytokine is known to recruit myeloid-derived suppressor cells (MDSCs) and regulatory T cells (Tregs)^27^. Even if causative genes themselves are different between human and rodents, further analyses for pathophysiological roles of identified genes would shed light on molecular basis of cardiovascular diseases.

## Methods

### Animal procedures

Seven subcongenic strains for both Chr-1 and Chr-18 QTLs were constructed from SHRSPrch1.1 and 18.0 through marker-assisted selection of pups with recombination in the target region as described previously^6^. The congenic region of each strain is shown in Fig. 2. A double subcongenic strain with both the Chr-1 and Chr-18 congenic regions was established from SHRSPrch1.1 and 18.0, and called SHRSPrch1.1_18.0 (Fig. 1c).

Stroke latency under salt-loading (1% salt in drinking water *ad libitum*) was evaluated as described previously^6^. Before salt-loading started, blood pressure (BP) was measured by the tail-cuff method.

BP measurement in SHRSPrch1.1_18.0 using the telemetry system was performed as described previously^6^. The average of systolic BP (SBP) between 11:00 and 12:00 represent SBP during the light phase (Fig. 1b). The same tendency was observed in SBP during the dark phase (represented by the average SBP between 23:00 and 01:00) (data not shown). All the procedures were reviewed and approved by the local committee for animal research at Shimane University. All the animal experiments were performed in accordance with relevant guidelines and regulations.

### Microarray analysis

The two parental strains, SHRSP and SHR, and the two original double congenic strains, SHRSPrch1_18 and SHRpch1_18^6^, were used in the experiment.

Five male rats at 12 weeks of age were either fed with plain water or with 1% salt water *ad libitum* for 1 week. Kidney was collected under deep anesthesia using diethyl ether and frozen in liquid nitrogen. The tissue samples were kept at −80 °C until analysis.

Whole kidney tissue was homogenized in Sepasol-RNA I Super G (Nacalai Tesque, Kyoto, Japan) using a Potter-Elvehjem tissue grinder and the supernatant was collected after centrifugation (1,000 rpm, 5 min at 4 °C). Total RNA was purified from an aliquot of the supernatant according to the manufacturer’s protocol followed by further purification using the NucleoSpin miRNA (Machery-Nagel, Düren, Germany). Extracted RNA was quantified on the Agilent 2100 Bioanalyzer (Agilent Technologies, Santa Clara, CA).

One hundred nanograms of total RNA was used to synthesize Cyanine 3-CTP labeled cRNA using the Low Input Quick Amp Labeling Kit, One-Color (Agilent Technologies) according to the manufacturer’s protocol. The labeled cRNA samples were hybridized on Rat GE 4 × 44 K v3 Microarray slides (Agilent Technologies) and hybridized slides were scanned with the SureScan Microarray Scanner (Agilent Technologies). Data quantification and quality control were performed using Feature Extraction 11.5.1.1 software (Agilent Technologies).

Normalization and subsequent data processing were performed using R software. In this procedure, the data sets of the control and salt-loaded group were separately standardized by the quantile normalization method, and then inter-strain differences were statistically examined. Probes showing ≥1.5 fold-difference with *P* < 0.05 (Student’s t-test) were considered significant for the purposes of screening. Expression of candidate genes was validated by quantitative RT-PCR as described previously^28^. PCR was performed on the 7300 Real Time PCR System (ThermoFisher Scientific, Waltham, MA) with a cycling condition as follows: 1 cycle of 95 °C for 30 s, followed by 40 cycles of 95 °C for 30 s and 60 °C for 60 s. Each sample was analyzed in duplicate. Specific amplification was confirmed by dissociation curve analysis. Primers used in the analysis are listed in Table S3.

### MicroRNA expression

Total kidney RNA from control or salt-loaded rats (n = 2, see Microarray analysis) were reverse-transcribed with TaqMan MicroRNA Reverse transcription Kit (ThermoFisher Scientific). Quantitative RT-PCR for miR-330-5p and miR-343 was performed using TaqMan RNA Assay (ThermoFisher Scientific) and Premix ExTaq Probe qPCR (Takara Bio, Shiga, Japan) on the 7300 Real Time PCR System (ThermoFisher Scientific). The PCR condition was as follows: 1 cycle of 95 °C for 30 s, followed by 40 cycles of 95 °C for 5 s and 60 °C for 30 s. U6 snRNA expression was used for standardization of miRNA expression levels. Each sample was analyzed in duplicate.

### Sequence analysis of candidate genes

Screening of coding variations was performed on previously obtained whole-genome sequence data of SHR and SHRSP^6^. Briefly, sequence reads were mapped on the Rattus Norvegicus genome assembly (ver. 6) using Bowtie (ver. 0.12.8), and variations in coding sequences were compared between the two strains using SAMtools (ver. 0.1.7).

Identified variations were confirmed by direct sequencing. Briefly, genomic PCR was performed with primer pairs as listed in Table S4. Amplified fragments were purified with QIAquick PCR Purification Kit (Qiagen, Hilden, Germany), and then used as a template for a sequencing reaction with BigDye Terminator v3.1 (ThermoFisher Scientific). DNA sequencing was performed on the ABI PRISM 3100 Genetic Analyzer (ThermoFisher Scientific). Genomic DNA from several rat strains was examined to determine whether observed variations were commonly present in laboratory rats. DNA (or liver tissue) of the rat strains used in the analysis were supplied by the National BioResource Project-Rat (http://www.anim.med.kyoto-u.ac.jp/NBR/).

Based on information of variations in the coding sequence, the 3D protein structure was built by submitting the amino acid sequence to I-TASSER (http://zhanglab.ccmb.med.umich.edu/), an online protein modeling server. In the analysis by I-TASSER, structural templates were first recognized from the Protein Data Bank (PDB) using multiple threading alignment approaches. Full-length structure models were then constructed by iterative fragment assembly simulations.

### Statistical analyses

Values are represented as mean ± SD. The Cox hazard model was employed for the analysis of stroke latency with BP (measured at 12 weeks of age by the tail cuff method) as a confounding factor. ANOVA and the Tukey’s post-hoc test were used to compare the gene expression level among different strains. JMP (v. 12, SAS) and Prism 7 (GraphPad Software Inc.) were used for the analyses. *P* < 0.05 was considered significant.

### Ethical approval

All the procedures of animal experiments were reviewed and approved by the local committee for animal research at Shimane University. All the animal experiments were performed in accordance with relevant guidelines and regulations.

## Electronic supplementary material

Supplementary information
