# Supplementary material for: Further dissection of QTLs for salt-induced stroke and identification of candidate genes in the stroke-prone spontaneously hypertensive rat
Source: Sci Rep. 2018 Jun 20;8:9403. doi: 10.1038/s41598-018-27539-2 (PMC6010461; doi:10.1038/s41598-018-27539-2)
Supplement: Supplementary file 1 — Supplementary information [file 41598_2018_27539_MOESM1_ESM.pdf]

Supplementary information

Further dissection of QTLs for salt-induced stroke and identification of candidate genes in the stroke-prone spontaneously hypertensive rat.

Kaoru Niiya[1], Hiroki Ohara[1*], Masato Isono[3], Abdullah Md.Sheikh[2], Hiroyuki Matsuo[1], Koichi Fujikawa[1], Minoru Isomura[4], Norihiro Kato[3], Toru Nabika[1]

[1)]Department of Functional Pathology and [2)]Department of Laboratory Medicine, Shimane University Faculty of Medicine, Izumo, Japan. [3)]Department of Gene Diagnostics and Therapeutics, Research Institute, National Center for Global Health and Medicine, Tokyo, Japan. [4)]Shimane University Faculty of Human Sciences, Matsue, Japan.

*To whom correspondence should be addressed to: Hiroki Ohara, Ph.D.

89-1 Enya-cho, Izumo, Shimane 693-8501, Japan

Telephone: +81-853-20-2407 FAX: +81-853-20-2135

E-mail: oharah@med.shimane-u.ac.jp

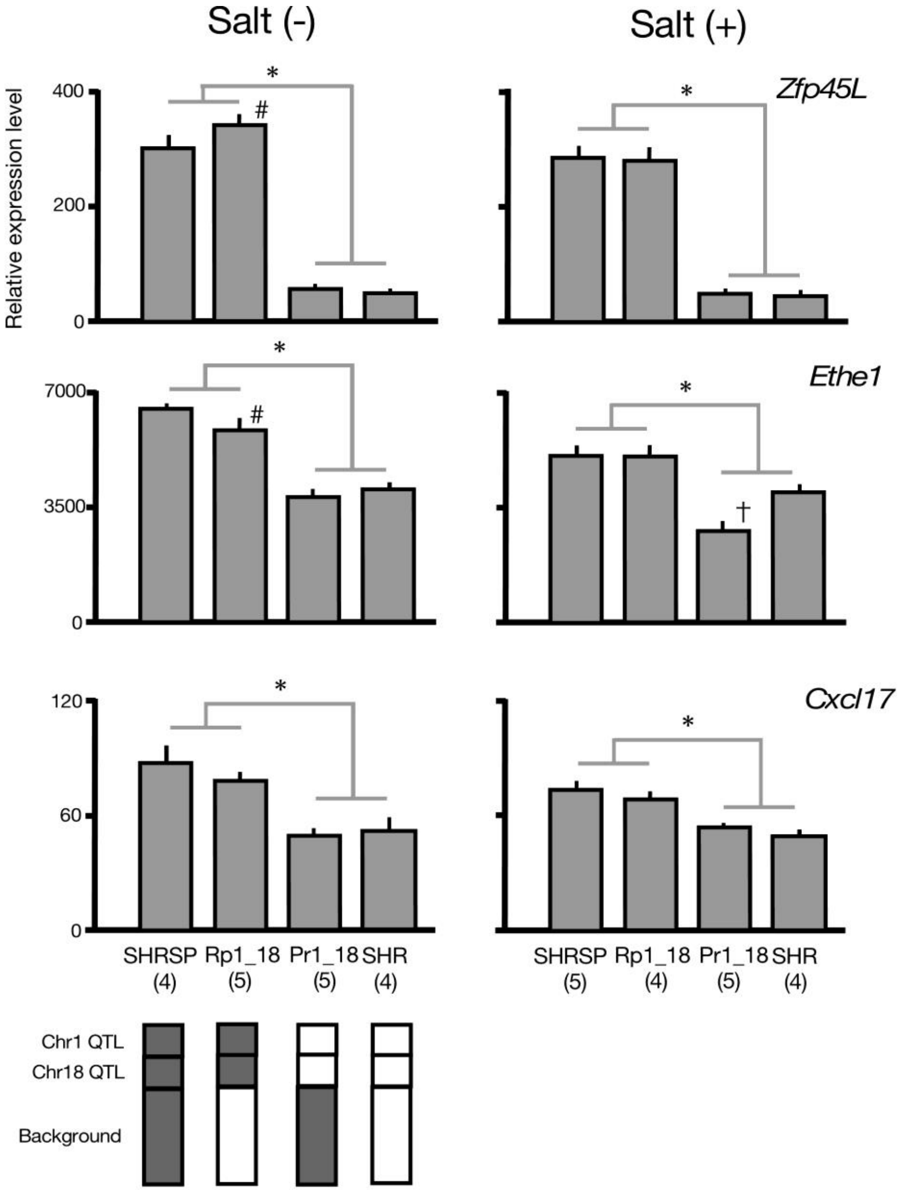

Figure S1. Gene expression of the three candidate genes by microarray analysis.

Kidneys were collected from rats with [Salt (+)] and without [Salt (-)] salt-loading (1% salt water for 1 week). Microarray analysis was performed as described in the Methods. SHRSPrch1_18 and SHRpch1_18 were abbreviated as Pr1_18 and Rp1_18, respectively. All three genes showed a significantly lower level of expression in both Pr1_18 and SHR than in Rp1_18 and SHRSP (*), which was consistent with the hypothetical expression pattern (see Results). In addition, significant differences were observed between SHRSP and Rp1_18 (#), and between SHR and Pr1_18 (†), in *Zfp45L* and *Ethe1*. Tukey's multiple comparison test was employed for the analysis. Numbers of rats used are in parentheses. Schematic patterns of genomic composition of the four strains are shown in the bottom of the figure; grey and open boxes indicate genomes from SHRSP and SHR, respectively.

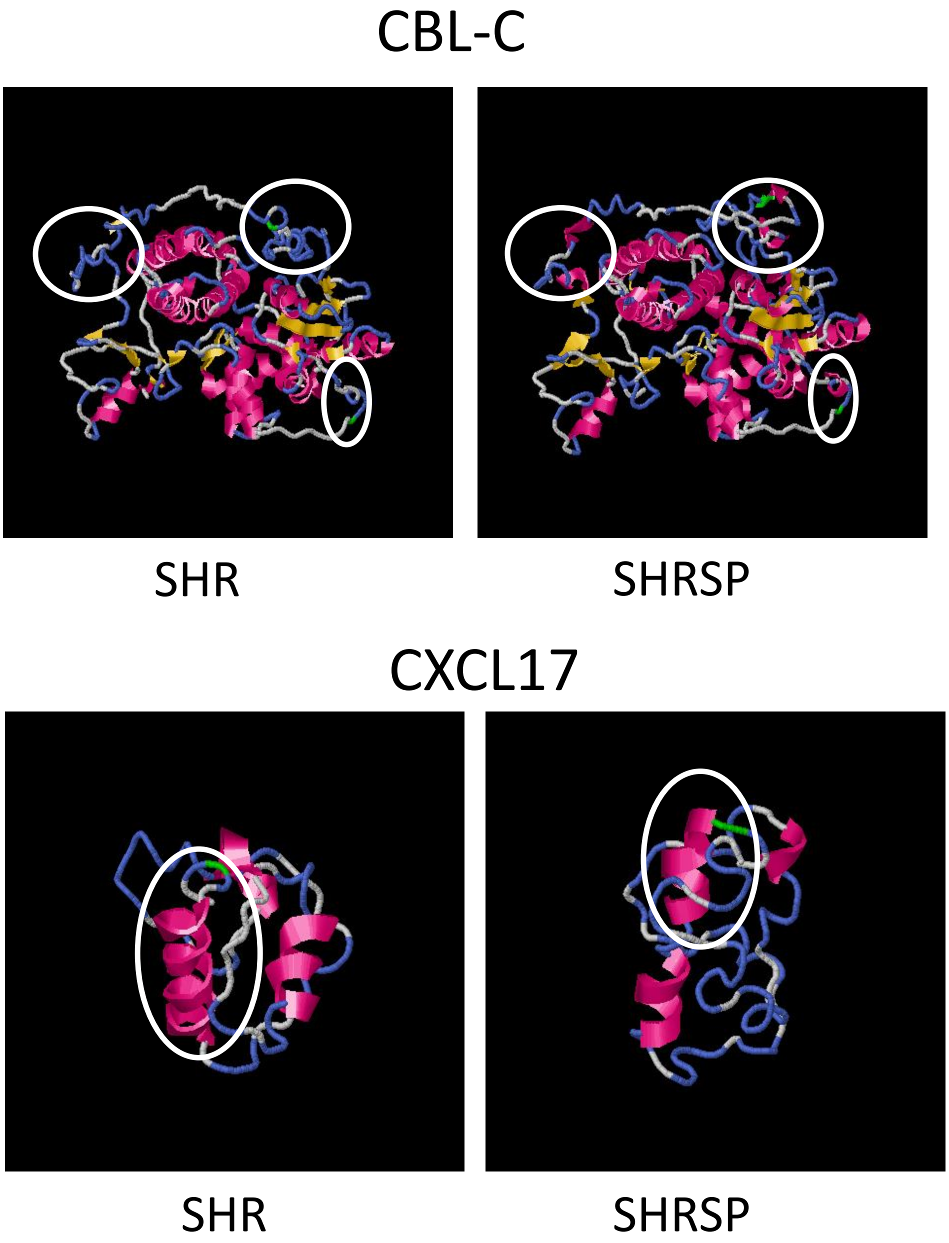

Figure S2. 3D-conformation of CBL-C and CXCL17 in SHR and SHRSP.

Analysis by I-TASSER implied substantial changes in the 3D-conformation of the proteins (shown with ovals). Pink; α-helix, Yellow; β-sheet, Blue; β-turn, Green; amino acids of mutation positions.

Table S1. Blood pressure and body weight of subcongenic strains.

(a) subcongcnic srains for the Chr-1 QTL and a double congenic strain for both the Chr-1 and -18 QTLs

	SP (27)	1.6 (23)	1.9 (22)	1.8 (28)	1.3 (27)	1.4 (47)	1.31 (20)	1.10 (23)	1.1 (18)	1.1_18.0 (20)
SBP, mmHg	194±10	205±11*	192±12	194±15	198±10	193±12	185±11*	189±9	194±11	186±13*
BW, g	265±25	267±14	254±12	261±15	267±26	268±18	277±20	281±26*	273±25	262±14

(b) subcongenic strains for the Chr-18 QTL

	SP (27)	18.1 (18)	18.2 (31)	18.3 (40)	18.4 (22)	18.6 (26)	18.7 (34)	18.8 (27)	18.0 (20)
SBP, mmHg	194±10	196±12	198±10	198±11	196±13	198±17	199±18	196±12	197±11
BW, g	265±25	252±15	265±29	252±17	255±15	261±14	261±14	261±22	261±18

Systolic blood pressure (SBP) was measured at 12 weeks of age by the tail cuff method. *: P<0.05 vs. SHRSP by Dunnet's

post-hoc test. Numbers of rats are in parentheses. ‘SHRSPrch’ is omitted from the names of subcongenic strains. SP: SHRSP.

Table S2. Direct sequencing in laboratory rat strains.

Strain	*Cblc*<br><br>Position in CDS: 1357 (exon 9)	*Cblc*<br><br>Position in CDS: 977 (exon 6)	*Cxcl17*<br><br>Position in CDS: 346 (exon 4)
SHRSP/A1-sb	G	A	G
SHR/Crj	A	T	A
WKY/Crj	A	T	A
Wistar	A	T	A
SR/JrNgs	A	T	A
SS/JrNgs	A	T	A
LEW/SsNSlc	A	T	A
F344/NSlc	A	T	A
DON/Kyo	G	A	G

Table S3. Primers used in RT-PCR.

Gene	forward	reverse
Ethe1	5' TGAACCCACGGCTCACTCTCAG 3'	5' CTGGACCCCACAGCGCATATTTG 3'
Zfp45L	5' CAGGAATATGAGGCGTGTGGGA 3'	5' CCCCGCACTCCTCACATGAATA 3'
Cxcl17	5' GATCATGTCAAGGGCAGTGAGA 3'	5' TATAAGGGCAGGGTGAAGCTTG 3'

Table S4. Primers used in direct sequencing.

Gene	forward	reverse
Cblc	5’ CCTGGTTGCTCTGGCTTTC 3’	5’ ACCCAACTCAGACCTCACTCAC 3’
	5’ ATGTACTCATGCACACACACCA 3’	5’ TCCAAGCCAGCACCACA 3’
Cxcl17	5’ ACAAAGGCCCTCCAGAAC 3’	5’ GGACAGAAGGCATGTAAG 3’
Ceacam19	5’ CAAAAGTCCTCCCTGAAACAACC 3’	5’ GGAGAAAGAGAGTGGATGAAAGACC 3’
Cic	5’ GCCTGTACCTCTGGGCATTCT 3’	5’ AGAGTGAAACGGATGCTGGTG 3’
